# Native kidney and graft survival in a cohort of Egyptian children with nephropathic cystinosis: national referral center experience

**DOI:** 10.1186/s13052-025-01943-7

**Published:** 2025-04-07

**Authors:** Rasha Helmy, Fatma M. Atia, Neveen A. Soliman

**Affiliations:** 1 Faculty of Medicine, Center of Pediatric Nephrology and Transplantation, Cairo University, Cairo, Egypt; 2 Cairo university Children Hospitals, Kasraliny medical school, Cairo university, Cairo, Egypt

**Keywords:** Cystinosis, Children, Outcome, CKD, Kidney transplantation

## Abstract

**Background:**

Nephropathic Cystinosis is a rare autosomal recessive lysosomal storage disorder. In addition to kidney dysfunction, this disorder can also affect other organs, such as the eyes, thyroid, muscles, and central nervous system.

**Methods:**

The current cross-sectional study included 56 patients with nephropathic cystinosis to evaluate the clinical outcome in nephropathic cystinosis patients cohort with regarding kidney function and the need for kidney replacement therapy. Clinical and laboratory data were collected.

**Results:**

Among the 56 patients in our study, 32 (57.1%) were male. Furthermore, 52 (92%) of these patients were offspring of consanguineous marriage. Patients’ mean age was 116.96 ± 54.1 months, and the mean onset of nephropathic cystinosis suggestive symptoms was 7.63 ± 3.2 months. In addition, the mean age of confirmed diagnosis was 45.38 ± 35.3 months, and the mean age of end-stage kidney disease (ESKD) was 104 ± 25.7 months. Eighteen patients (32.1%)underwent hemodialysis, whereas 12 patients (21.4%) underwent kidney transplantation. When comparing siblings within the same family, we observed a significant difference in the age at diagnosis. The median age for the first sibling was 60 months, while it was 24 months for the second sibling (*p*-value = 0.031). Additionally, there were significant differences in weight, chronic kidney disease (CKD) stage, and outcome.

**Conclusion:**

Improvement in the awareness and the accessibility to diagnosis over years, early sibling screening, and kidney transplantation have a significant impact on the survival of both patients and kidney in children with nephropathic cystinosis.

## Background

Nephropathic Cystinosis (NCTN)is a rare autosomal recessive lysosomal storage disorder caused by a genetic variant in the *CTNS* gene. It is characterized by the accumulation of intra-lysosomal cystine in all body cells and organs, leading to kidney impairment and affection of various organs, including eyes, thyroid, muscles, and the central nervous system (CNS) [1].

Fanconi syndrome is a proximal tubular dysfunction that manifests in the first year of life. It leads to polydipsia, polyuria, dehydration, proximal renal tubular acidosis, and urinary loss of electrolytes. If left untreated, ESKD affects the median age of about ten years [2].

Although cysteamine, a cystine-depleting agent, is the cornerstone of treatment, no curative treatment is currently available [1]. Numerous reports have demonstrated favorable graft performance and outcomes in patients with NCTN [3, 4].

Prior to kidney transplantation and cysteamine, individuals with nephropathic cystinosis had a lifespan of around 10 years. However, they have now managed to survive into their fifth decade [5].

The current study focuses on assessing kidney prognosis and patient survival in a cohort of Egyptian patients with nephropathic cystinosis.

## Methods

### Study design and population

This cross-sectional study included 56 patients with NCTN. The patients were diagnosed with NCTN using slit lamp examination for cystine deposition or genetic analysis. Study participants were recruited from the multidiciplinary Cystinosis Clinic at Cairo University Children’s Hospital which is the only national referral center.

### Sample size

This study included 56 patients following at the cystinosis clinic, ( the total number registered at the clinic was 77 patients from 2003 to 2023);We excluded 21 patients due to the unavailability of complete medical records and lost follow up.

### Clinical assessment

Full clinical assessment focusing on the current age, age at onset of symptoms, age at confirmed diagnosis, family history, consanguinity, anthropometric measurement, kidney replacement therapy (KRT), age at kidney replacement, ophthalmological assessment, medications received, and cysteamine dose. The weight and height parameters were standardized using z-scores, calculated based on reference data from healthy children. These parameters were then interpreted according to world health organization (WHO) curves [6].

### Reviewing of previous investigations

Blood urea nitrogen, serum creatinine, calcium, phosphorus, alkaline phosphatase, venous blood gases, complete blood count, serum albumin, and serum electrolytes tests were reviewed. The staging of CKD was determined according to the estimated glomerular filtration rate (eGFR) [ml/min/1.73 m2] as follows: stage 3 (eGFR = 30–59), stage 4( eGFR = 15–29), and stage 5 (eGFR < 15). In addition, eGFR was assessed according to the modified Schwartz formula [7, 8].

### Statistical analysis

Data were analyzed, coded, and analyzed using IBM-SPSS 24.0 (IBM-SPSS Inc., Chicago, IL, USA) *. Descriptive statistics: Means, standard deviations, median, Interquartile range, frequency, and percentages were calculated. The normality of continuous variables was tested using the Kolmogorov–Smirnov/Shapiro–Wilk test as appropriate. Test of significances: Chi-square/Fisher’s exact/Monte Carlo exact test was used to compare the difference in the distribution of frequencies among different groups as appropriate. Student t-test/Mann Whitney U-test was calculated to test the mean/median differences in continuous variables between groups as appropriate. Kruskal Wallis test was used to compare the difference in the median between groups for variables with more than two categories. The Spearman Rank Correlation was used to analyze the univariate correlation between variables. Significant test results were considered when the p-value was < 0.05.

## Results

This study included 56 patients with nephropathic cystinosis, with 32 (57.1%) males and 24 (42.9%) females. Of the patients, 52 (92.9%) were born to consanguineous parents, while 34 (60.7%) had a family history of similar conditions. The mean age at diagnosis was 45.38 months (SD ± 35.3) with median 35 months ( range 3 – 168).The time elapsed between the onset of symptoms and the confirmation of diagnosis ranged from 0- 156 months with mean 37.27 ± 34.6 months.

Basic laboratory data of the cohort were shown in Table 1.

**Table 1:** Laboratory data of the studied Cohort (*n* = 56)

Variable	Category	*n* = 56
Kidney functions		
**Blood Urea (mg/dl)**	• **Mean ± SD**	72.32 ± 66.7
	• **Median (Range)**	46 (6 – 250)
**Serum Creatinine (mg/dl)**	• **Mean ± SD**	2.99 ± 2.7
	• **Median (Range)**	1.1 (0.3 – 9.3)
**e-GFR**	• **Mean ± SD**	37.61 ± 33.1
	• **Median (Range)**	36 (4 – 120)
**Serum Electrolytes**		
**Ca (mg/dl)**	• **Mean ± SD**	8.96 ± 1.7
	• **Median (Range)**	9.6 (5.3 – 11.5)
**PO**_**4**_** (mg/dl)**	• **Mean ± SD**	4.20 ± 1.1
	• **Median (Range)**	4 (2 – 9)
**Na (mmol/L)**	• **Mean ± SD**	139.01 ± 6.5
	• **Median (Range)**	139 (130 – 156)
**K (mmol/L)**	• **Mean ± SD**	3.92 ± 1.1
	• **Median (Range)**	3.8 (2 – 6)
**CBC**		
**Hgb (g/dl)**	• **Mean ± SD**	10.38 ± 2.5
	• **Median (Range)**	11 (6 – 14)
**MCV(fl)**	• **Mean ± SD**	79.42 ± 6.2
	• **Median (Range)**	79.5 (69 – 90)
**MCH(pg)**	• **Mean ± SD**	26.61 ± 2.1
	• **Median (Range)**	27 (23 – 29.5)
**Hct (%)**	• **Mean ± SD**	30.44 ± 6.6
	• **Median (Range)**	32 (18 – 40)
TLC(10*3/cmm)	• **Mean ± SD**	8.38 ± 3.2
	• **Median (Range)**	7.5 (2.5 – 14.5)
Platelet(10*3/cmm)	• **Mean ± SD**	309.06 ± 105.2
	• **Median (Range)**	323 (54 – 532)
**Other Parameters**		
**ALP(U/l)**	• **Mean ± SD**	462.53 ± 328.9
	• **Median (Range)**	340 (138 – 1277)
**Albumin(g/dl)**	• **Mean ± SD**	4.31 ± 0.7
	• **Median (Range)**	4.5 (2.8 – 5.1)
**PH**	• **Mean ± SD**	7.36 ± 0.1
	• **Median (Range)**	7.4 (7.2 – 7.5)
**Bicarbonate (mmEq/l)**	• **Mean ± SD**	20.91 ± 6.6
	• **Median (Range)**	21 (10 – 32)

*e-GFR* Estimated glomerular filtration rate, *Ca* Calcium, *PO4* Phosphorus, *Na* Sodium, *K* Potassium, *Hbg* Haemoglobin, *MCV* mean corpuscular volume, *MCH* mean corpuscular haemoglobin, *Hct* hematocrite, *TLC* total leukocytic count, *ALP* alkaline phosphatase

The presenting symptoms included either renal tubular acidosis 47 (83.9%) or rickets 8 (14.3%), while one of our patients (1.8%) was diagnosed presymptomatic during screening using genetic testing; he is now 10 years old manifesting only mild acidosis and hypophosphatemia that started a year ago. The mean weight of the included patients was 19.24 ± 9.1 kg. Out of the patients, 45 (80.4%) were classified as underweight below −2 SD for *z-*score, while the mean height was 104.81 ± 19.7 cm. Additionally, 50 (89.3%) patients were stunted below −2SD for z -score, and 35 (35/50) were below −4 SD. Five patients received growth hormone therapy. However, it was discontinued for 4 of them due to inaccessibility (1/5), acute rejection episode (1/5), papilledema(1/5), or bone deformity(1/5).

Hypothyroidism was managed with L-thyroxin replacement therapy in 38 (67.9%) of the patients. The majority (67.8%) of these patients received cysteamine treatment at doses ranging from 45 to 60 mg/kg/d. Nevertheless, a total of 18 individuals (32.2%) failed to adhere to or discontinued their treatment. In addition, 16.1% of our patients did not attend their follow-up appointments, while 10 patients (17.9%) passed away at a mean age of 111.10 ± 45.6 months. The causes of death were complications related to hemodialysis or severe electrolyte disturbances and hypovolemic shock.

Regarding CKD staging of our patients, 6 patients (10.7%) were classified as stage 1, 7 patients (12.5%) were classified as stage 2, 12 patients (21.4%) were classified as stage 3, 7 patients (12.5%) were classified as stage 4, and 24 patients (42.9%) were classified as stage 5. The mean age of patients diagnosed with ESKD was 104 ± 25.7 months with median age 96 months ranged from 74 to 162 months. Among the total number of patients, 18 (32.1%) received Hemodialysis (HD) treatment, while 12 patients (21.4%) underwent kidney transplantation (KTX).

Out of the 12 KTX patients,all of them received living grafts, 7 received kidney grafts from unrelated donors, 4 received grafts from their mothers, and one child received a graft from his father. The duration post kidney transplantation ranged from 7 to 180 months.the eldest of our transplanted cases is 25 years old with a graft duration of 13 years and her last serum creatinine level is 1.3 mg/dl. Only one of the transplanted cases in our cohort died of severe infection. All of them received only one graft and they were on maintenance immunosuppressive protocol consisting of steroid, tacrolimus, and mycophenolate except one female child who was on steroid avoidance protocol with serum creatinine level of 0.5 mg/dl. Data of transplanted patients were demonstrated in Table 2.

**Table 2:** Summary of the transplanted patients in the studied cohort (*n* = 12)

No	Age (months)	Donor	Duration of transplantation (months)	Height z score	Weight Z score	Last creatinine
1	300	Living related (mother)	156	< −2	< −2	1.3
4	176	Living related (father)	56	< −2	< −2	0.9
7	163	Living unrelated	38	< −2	−1	0.7
13	167	Living unrelated	67	< −2	< −2	1.1
17	190	Living unrelated	36	< −2	< −2	0.4
20^a^	150	Living unrelated	7	< −2	< −2	2.3
22	121	Living unrelated	44	−1.2	−0.5	0.48
23	168	Living unrelated	69	< −2	< −2	0.73
26	130	Living unrelated	11	< −2	0	0.86
27	97	Living related (mother)	14	−1.7	−0.2	0.64
28	176	Living related (mother)	33	< −2	< −2	0.55
31	140	Living related (mother)	49	< −2	< −2	0.6

^a^This case died after 7 months of transplantation due to severe infection

**Fig. 1:**
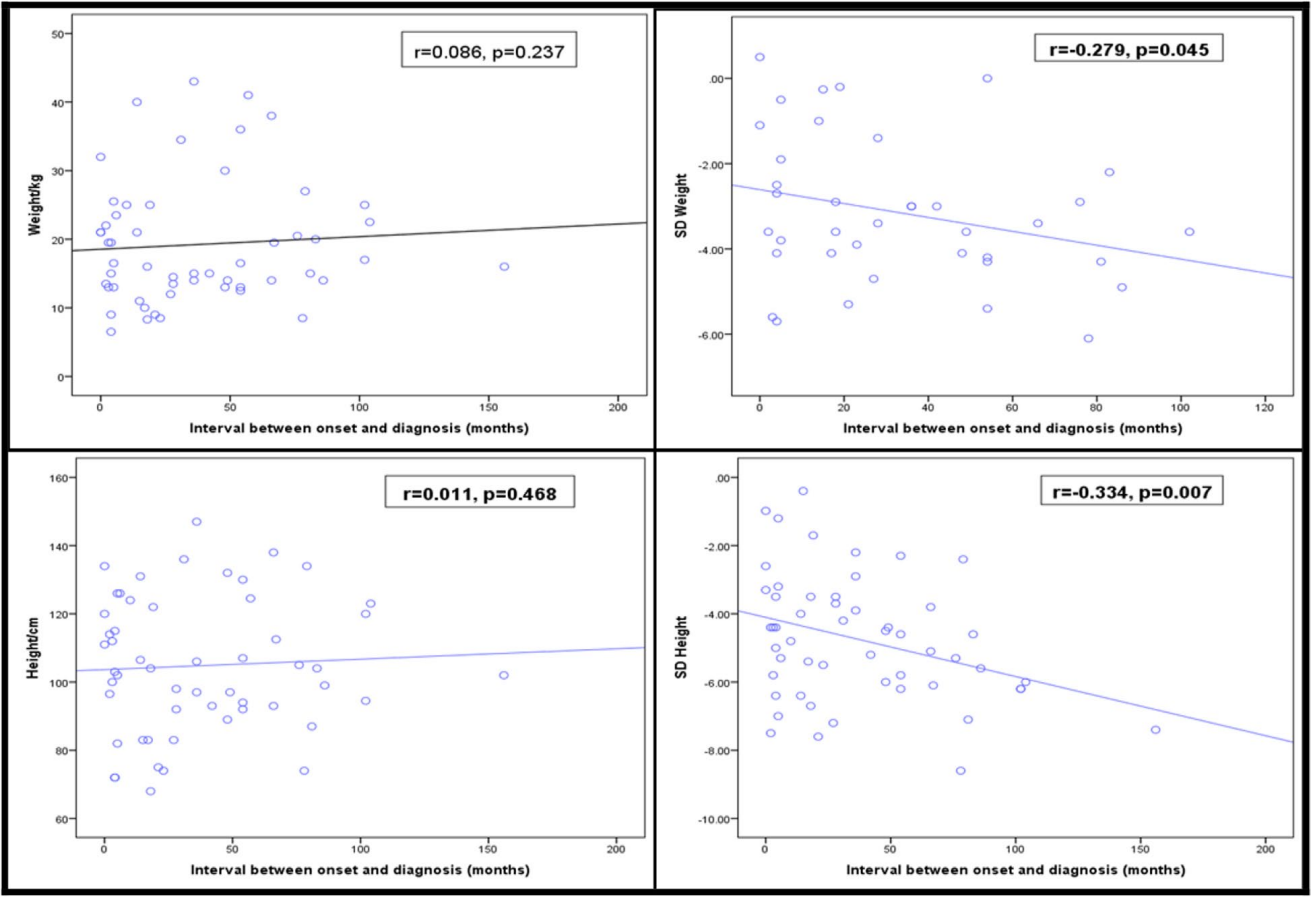
Correlation between onset to diagnosis interval and anthropometric measures. SD: standard deviation for z-scores of weight and height. **A** showed non significant correlation between onset-diagnosis interval and weight in kilograms; **B** showed non significant correlation between onset -diagnosis interval and height in centimeters; **C** showed significant correlation between onset-diagnosis interval and Z score for weight; **D** showed significant correlation between onset-diagnosis interval and Z score of height

When we correlated the absolute values for weight and height with the onset -diagnosis interval,no statistically signigicant relationship was found.However, the longer duration between the onset of symptoms and confirmed diagnosis -subsequently; the start of treatment – was associated with statistically signigicant lower z -scores for both weight and height, as demonstrated in Fig. 1. However, no significant correlation was observed between CKD staging in the patients or the disease outcome, as depicted in Table 3.

**Table 3:** Relationship between onset to diagnosis interval, CKD Stage and disease outcome

	**CKD Stage**					***P*-value**
	**Stage-I** **(*n* = 6)**	**Stage-II** **(*n* = 7)**	**Stage-III** **(*n* = 12)**	**Stage-IV** **(*n* = 7)**	**Stage-V** **(*n* = 24)**	
•onset to diagnosis interval /months **Median (IQR)**	35.5 (81)	28 (40)	24.5 (30)	27 (62)	36 (76)	= 0.750*
	**Disease Outcome**					***P*-value**
	**Alive** **(*n* = 37)**		**Death** **(*n* = 10)**	**Lost FU** **(*n* = 9)**		
•onset to diagnosis interval /months **Median (IQR)**	28 (42)		21.5 (53)	54 (80)		= 0.571*

*IQR* Interquartile range

^*^Kruskal Wallis test was used to compare the difference in median between groups

The mean corneal cystine crystal scoring (CCCS) of our patients was 2.63 ± 0.7. For patients with hypothyroidism, the average CCCS was 2.84 ± 0.4, while for patients with euthyroidism, it was 2.21 ± 0.2. There was a significant difference between the two groups, with a p-value of 0.013.

The comparison between patients who underwent hemodialysis (HD) and those who underwent kidney transplantation revealed a significant difference in the anthropometric measurement (Table 4).

**Table 4:** Comparison between kidney replacement modalities and anthropometric measurements

	**KRT**		
	**KTX (*n* = 12)**	**HD (*n* = 18)**	***P*-value**
• **Weight/kg**	32.04 ± 7.7	18.47 ± 4.1	< 0.001*
• **Underweight (< −2 SD)**	8 (66.7%)	18 (100%)	= 0.019**
• **Height/cm**	129.63 ± 8.8	106.16 ± 13.4	< 0.001*
• **Stunted (< −2 SD)**	10 (83.3%)	18 (100%)	= 0.165**
• **CKD Stage**			
✓ **Stage-I**	4 (33.3%)	0 (0%)	
✓ **Stage-II**	5 (41.7%)	0 (0%)	< 0.001***
✓ **Stage-III**	2 (16.7%)	0 (0%)	
✓ **Stage-IV**	0 (0%)	0 (0%)	
✓ **Stage-V**	1 (8.3%)	18 (100%)	

*CKD* Chronic kidney disease, *KRT* Renal replacement therapy, *k-tx* Kidney transplantation, *HD* hemodialysis

^*^Independent Sample t-test was used to compare the difference in mean between groups

^**^Chi-square test was used to compare the difference in frequency between groups

^***^Monte Carlo exact test was used to compare the difference in frequency between groups

In this cohort, we observed 22 siblings. We conducted a subgroup analysis comparing the first sibling (1st sibling) to the second sibling (2nd sibling). We discovered a significant difference in the age at diagnosis between the two groups. The median age at diagnosis for the first sibling was 60 months, while it was 24 months for the second sibling, with a p-value of 0.031). Furthermore, there was a significant difference regarding weight, CKD stage, and outcome, as illustrated in Table 5.

**Table 5:** Relationship between birth order and disease determinants

	**Siblings**		***P*-value**
	**1st sibling** **(*n* = 11)**	**2nd sibling** **(*n* = 11)**	
**Age at diagnosis/months**			
• **Mean ± SD**	48.64 ± 29.9	29.27 ± 20.1	= 0.031*
• **Median (IQR)**	60 (56)	24 (26)	
**Anthropometrics**			
• **Underweight**	8 (88.9%)	6 (54.5%)	= 0.026**
• **Stunted**	11 (100%)	9 (81.8%)	= 0.479**
**CKD stage**			
• **Stage-I**	1 (9.1%)	1 (9.1%)	
• **Stage-II**	2 (18.2%)	3 (27.3%)	
• **Stage-III**	2 (18.2%)	3 (27.3%)	= 0.299***
• **Stage-IV**	0 (0%)	2 (18.2%)	
• **Stage-V**	6 (54.5%)	2 (18.2%)	
**Outcome**			
• **Alive**	5 (45.5%)	10 (91.9)	
• **Dead**	6 (54.5%)	0 (0%)	= 0.013***
• **Lost follow up**	0 (0%)	1 (9.1%)	

*IQR* Inter-Quartile range, *SD* Standard deviation

^*^Mann Whitney U test was used to compare the difference in median between groups

^**^Fisher’s exact test was used to compare the difference in Frequency between groups

^***^Monte Carlo exact test was used to compare the difference in Frequency between groups

## Discussion

The most significant finding of our study is that the second siblings diagnosed at an earlier age exhibited better outcomes compared to their older siblings in terms of the age at diagnosis,weight, and overall outcome. This finding aligns with a previous study [9]. This finding can be attributed to the early intervention of either Fanconi syndrome replacement therapy or cysteine-depleting therapy.

In our cohort, we had one patient diagnosed as presymptomatic using sibling screening based on genetic variant analysis at the age of 3 months when he started cystasteamine therapy. He is 10 years old with growth parameters within the normal range ( −0.98 SD for height and 0.5 SD for weight) and mild tubular dysfunction. Additionally, his eGFR was 138 ml/min/1.73 m2. This discovery highlights the significance of screening siblings and providing early patient treatment, as suggested by a previous review [1].

This signifies the importance of early diagnosis and therapy for those patients. In our country,as developing and middle income one,there were a lot of chalanges for the care of our patients with NCTN in the form of low accessibility to genteic testing, unavailability of white blood cell cysteine assay and inpersistency of cysteine depleting therapy, We have overcome these challenges over the past years, and this has been reflected on patients in terms of doctors’ and families’ awareness of the disease, referral of cases at an early stage, as well as the availability of persistent treatment. When comparing the current study to its previous in the same center, we notice the mean age at diagnosis became earlier ( 45.38 vs 52.7 months in the previous study) [10].

In addition, most of our children on average dose of cystemine treatment of 45–60 mg/kg/day with improvement in daily dosage,patient compliance and the persistency of treatment in comparision to previous study in the same center [11].

The factors that contribute to growth failure in children with NCTN include uremia, inadequate cystine-depleting therapy, rickets, chronic acidosis, anemia, and chronic hypovolemia [12].

In our cohort, we observed a high prevalence of underweight (below −2 SD) and stunted growth (below −2 SD), with percentages of 80.4% and 89.3% respectively. These findings are consistent with previous results from the same center. However, these results are inconsistent with *Kluck and his colleagues* [13], who demonstrated the mean SD for height _1.80 (_2.05 to _1.55) and the mean SD for body mass index _0.28 (_0.55 to _0.02). The severe impairment of growth parameters in our cohort in comparison to their cohort can be explained by the fact that they used growth hormone therapy(GHT) for about half of the patients and tube feedings if indicated. In contrast, only five patients in our cohort received GHT, and no tube feedings were required. It is important to note that this difference cannot be solely explained by malnutrition, as most of our study participants had only mild anemia and normal serum albumin levels. These findings highlight the importance of growth hormone therapy, as concluded by *Wüh and his colleagues* [14] and also highlight the importance of multidisciplinary care involving endrinology,nutrition and other involved specialities.

The mean eGFR at the last visit was 37.61 ± 33.1 ml/min/1.73 m2, which is lower than the eGFR reported in another study, 63.10 ± 54.60 ml/min/1.73 m2 [15]. *Atmis and his colleagues* [15] found that the median age at diagnosis was 18.5 months (range, 6–205 months), and in another study [16] was 15 months ( range,0–110). In contrast, the median age at diagnosis of our patients was 35 months (range,3–168). The delay could be attributed to a lack of awareness and limited availability of diagnostic tools for nephropathic cystinosis especially the genetic testing before detection of cysteine deposion in the cornea that could be delayed until the age of 18 months. Additionally, the median time between the onset of the disease and a confirmed diagnosis was 28 months (with a range of 0–156). Therefore, patients were referred to receive treatment at a late stage when their kidney has reached an advanced stage of dysfunction.

Out of the total number of patients, 30 patients (53.5%), 14 female and 16 male, underwent KRT (either dialysis or kidney transplantation). The mean age at initiation of KRT was 104 ± 25.7 months and median of 96 months ( range: 74–162), which is lower than the median age reported in previous studies [16, 17], in which the median age at initiation of KTX was 120 months (range: 84–300). Potentially, this could be attributed to a delayed diagnosis in our group. However, we did not detect any significant correlation between the stage of CKD and the time interval between the onset and diagnosis (p-value = 0.75), in contrast to what demonstrated by Emma et al., who found a nearly linear relationship between the age at which cysteamine therapy commenced and the outcome of kidney function [12].

Kidney transplantation enhanced the growth parameters of our children when compared to those undergoing regular hemodialysis. There was a notable difference in terms of weight and height, which aligns with a previous study that found an improvement in linear growth after kidney transplantation [18]. However, there was still a higher percentage of children experiencing stunted growth. Analysis of data from registries in Europe and North America indicates that a functional graft alone does not rectify the significant growth retardation observed in some children with CKD. It is recommended to implement more proactive management strategies before and after transplantation and consider using GHT [19].

Prior to kidney transplantation and cystine-depleting therapy with cysteamine for children with nephropathic cystinosis, their lifespan was approximately ten years. Currently, cystinotic patients have survived until the fifth decade of life [5]. The mortality rate in our cohort was 17.9% of the patients, with a median age of death of 114.5 months (range 16–180). The majority of mortality was attributed to electrolyte disturbances and complications related to hemodialysis, such as pulmonary edema. None of these patients received continuous cysteamine treatment.

Thyroid dysfunction is the most common and earliest endocrine disorder observed in patients with cystinosis, affecting approximately 50% of untreated children and typically occurring after kidney dysfunction [20]. Hypothyroidism was prevalent in 38 (67.9%) of the patients, which is consistent with previous research [21]. All patients were on hormonal replacement therapy. This higher prevalence could be suggested to be secondary to the delayed diagnosis as well as the inconsistent use of cysteamine treatment.

Furthermore, we found a significant difference in CCCS between patients with hypothyroidism and those with euthyroidism. This discrepancy may be attributed to the age of the patients, as younger patients exhibited preserved thyroid function and lower cysteine accumulation in the cornea.

The high consanguinity rate of 92% in our cohort can be attributed to the autosomal recessive mode of disease inheritance and the prevalence of marriage between relatives in our society. This result is in contrast to a study conducted in Poland, where the consanguinity rate was 0% [17].

## Conclusion

Improvement in the awareness and accessibilty to diagnosis over years help in early diagnosis, particularly pre-symptomatic, and thus initiation of cysteine depleting therapy which is crucial to kidney and patient survival in nephropathic cystinosis patients. In patients reaching ESKD, Kidney transplantation significantly improved the patients’ clinical outcome with graft survival reaching up to 13 years in our cohort. Moreover integrated approach in the context of multidisciplinary care is essential to ensure comprehensive management and optimize patient growth.

Recommendations: we recommend increasing the awareness among young healthcare providers regarding early detection and referral of infants with Fanconi syndrome to facilitate the prompt diagnosis of this manageable condition as earlier treatment improves disease outcome. Additionally, multidisciplinary care and further studies of long-term follow up post kidney transplantation outcome are recommended of those patients.

## Data Availability

The datasets generated during and/or analyzed during the current study are available from the corresponding author upon reasonable request.

## Abbreviations

CCCS: Corneal cystine crystal scoring
CKD: Chronic kidney disease
CNS: Cenral nervous system
eGFR: estimated glomerular filtration rate
ESKD: End stage kidney disease
GHT: Growth hormone therapy
HD: Hemodialysis
KRT: Kidney replacement therapy
KTX: Kidney transplantion
NCTN: Nephropathic cystinosis
WHO: World health organization

## References

[1] Elmonem MA, Veys KR, Soliman NA, Van Dyck M, Van Den Heuvel LP, Levtchenko E. Cystinosis: A review. Orphanet J Rare Dis. 2016;11(1):1–17. Available from: 10.1186/s13023-016-0426-y 10.1186/s13023-016-0426-y PMC4841061 27102039

[2] Bäumner S, Weber LT. Nephropathic cystinosis: Symptoms, treatment, and perspectives of a systemic disease. Front Pediatr. 2018;6(March):1–8. 29594088 10.3389/fped.2018.00058 PMC5861330

[3] El Ghoul K, Akiki D, Nawfal N, Jaoude MA. Renal transplantation for infantile and juvenile cystinosis: Two case report and review of the literature. Transpl Immunol. 2024;83:101993. Available from: https://www.sciencedirect.com/science/article/pii/S0966327424000091 10.1016/j.trim.2024.101993 38224843

[4] Gheith O, Nair P, Adel M, Adel M, Denewar A, Mahmoud T, et al. Cystinosis in Pediatric Renal Transplant Recipients: A Case-Control Study From Kuwait. Exp Clin Transplant Off J Middle East Soc Organ Transplant. 2022;20(Suppl 1):95–9. 10.6002/ect.MESOT2021.P40 35384816

[5] Nesterova G, Gahl W. Nephropathic cystinosis: late complications of a multisystemic disease. Pediatr Nephrol. 2008;23(6):863–78. 18008091 10.1007/s00467-007-0650-8

[6] Physical status: the use and interpretation of anthropometry. Report of a WHO Expert Committee. Vol. 854, World Health Organization technical report series. Switzerland; 1995. 8594834

[7] Inker LA, Astor BC, Fox CH, Isakova T, Lash JP, Peralta CA, et al. KDOQI US commentary on the 2012 KDIGO clinical practice guideline for the evaluation and management of CKD. Am J kidney Dis Off J Natl Kidney Found. 2014;63(5):713–35. 10.1053/j.ajkd.2014.01.416 24647050

[8] Schwartz GJ, Brion LP, Spitzer A. The use of plasma creatinine concentration for estimating glomerular filtration rate in infants, children, and adolescents. Pediatr Clin North Am. 1987;34(3):571–90. 3588043 10.1016/s0031-3955(16)36251-4

[9] Veys K, Zadora W, Hohenfellner K, Bockenhauer D, Janssen MCH, Niaudet P, et al. Outcome of infantile nephropathic cystinosis depends on early intervention, not genotype: A multicenter sibling cohort study. J Inherit Metab Dis. 2022;(August):1–12. 10.1002/jimd.12562 36117148

[10] Soliman NA, El-Baroudy R, Rizk A, Bazaraa H, Younan A. Nephropathic cystinosis in children: An overlooked disease. Saudi J kidney Dis Transplant an Off Publ Saudi Cent Organ Transplantation, Saudi Arab. 2009;20(3):436–42. 19414947

[11] Soliman NA, Bazaraa HM, Abdel Hamid RH BN. of Kidney Diseases and Transplantation Letter to the Editor Nephropathic Cystinosis in a Developing Country : The Egyptian Experience. 2013;24(1):147–9. 10.4103/1319-2442.106315 23354215

[12] Emma F, Hoff WV, Hohenfellner K, Topaloglu R, Greco M, Ariceta G, et al. An international cohort study spanning five decades assessed outcomes of nephropathic cystinosis. Kidney Int. 2021;100(5):1112–23. 34237326 10.1016/j.kint.2021.06.019

[13] Kluck R, Müller S, Jagodzinski C, Hohenfellner K, Büscher A, Kemper MJ, et al. Body growth, upper arm fat area, and clinical parameters in children with nephropathic cystinosis compared with other pediatric chronic kidney disease entities. J Inherit Metab Dis. 2022;45(2):192–202. 34989402 10.1002/jimd.12473

[14] Wühl E, Haffner D, Offner G, Broyer M, Hoff WV t., Mehls O. Long-term treatment with growth hormone in short children with nephropathic cystinosis. J Pediatr. 2001;138(6):880–7. 10.1067/mpd.2001.113263 11391333

[15] Atmis B, K. Bayazit A, Cevizli D, Kor D, Fidan HB, Bisgin A, et al. More than tubular dysfunction: cystinosis and kidney outcomes. J Nephrol. 2022;35(3):831–40. Available from: 10.1007/s40620-021-01078-y 10.1007/s40620-021-01078-y 34097292

[16] O’Connell N, Oh J, Arbeiter K, Büscher A, Haffner D, Kaufeld J, et al. Patients With Infantile Nephropathic Cystinosis in Germany and Austria: A Retrospective Cohort Study. Front Med. 2022;9(April):1–10. 10.3389/fmed.2022.864554 PMC9082678 35547226

[17] Sikora P, Grenda R, Kowalczyk M, Kieć-Wilk B, Bieniaś B, Rubik J, et al. Nephropathic cystinosis in Poland: a 40-year retrospective study. Polish Arch Intern Med. 2022;132(11):1–9. 10.20452/pamw.16320 35997069

[18] Lopez-Gonzalez M, Munoz M, Perez-Beltran V, Cruz A, Gander R, Ariceta G. Linear Growth in Pediatric Kidney Transplant Population. Front Pediatr. 2020;8(December):1–7. 33364221 10.3389/fped.2020.569616 PMC7752780

[19] Drube J, Wan M, Bonthuis M, Wühl E, Bacchetta J, Santos F, et al. Clinical practice recommendations for growth hormone treatment in children with chronic kidney disease. Nat Rev Nephrol. 2019;15(9):577–89. 31197263 10.1038/s41581-019-0161-4 PMC7136166

[20] Gahl WA, Thoene JG, Schneider JA. Cystinosis. N Engl J Med. 2002;347(2):111–21. 12110740 10.1056/NEJMra020552

[21] Algasem R, Zainy N, Alsabban E, Almojalli H, Raza S, Ali T, et al. The Clinical Manifestations and Disease Burden of Cystinosis in Saudi Arabia: A Single-Tertiary Center Experience. Cureus. 2024;16(1):1–12. 10.7759/cureus.52662 PMC10877213 38380220

